# Thank you to our Reviewers!

**DOI:** 10.1016/j.ebr.2021.100448

**Published:** 2021-03-26

**Authors:** William O. Tatum IV, D.O.

**Affiliations:** Mayo Clinic College of Medicine & Health Science, Jacksonville, FL, US

During an unprecedented time, Epilepsy & Behavior Reports (EBR) has experienced an unprecedented year. We have seen a significant increase in the number of high-quality submissions during the pandemic. In addition, the number of case series, and retrospective (some prospective) studies have been published advancing our goal of becoming a premier global epilepsy journal available on the world-wide web via public (open) access. Information on clinical epilepsy, surgical therapy, new antiseizure medication, semiology, intracranial EEG, neuropsychological assessment, and social issues including SUDEP have been included in accepted journal articles. Multiple disciplines have involved neurology, neurosurgery, neuroradiology, neuropsychologic, neurophysiology, psychiatry/psychology, and pathology from epilepsy centers, communities, and rural areas from countries and territories from all over the world.

In the last year, EBR has grown with the addition of an editorial board overseeing different aspects of our peer-reviewed journal that boasts an average of 2 reviews/submission. A special thanks to Drs. Jerzy Szflarski, Kurupath Radhakrishnan, Barry Gidal, Arthur Grant, and Sallie Baxendale for always setting the bar high to augment what EBR has to offer. Multiple publication types are now available including novel case reports and case series, full-length manuscripts, review articles, short communications, and letters to the editor in the form of lay abstracts, enhancements, topical issues, and more. Our international list of reviewers has been amazing with a willingness to ensure quality manuscripts become available in the many countries and territories throughout the world. It is with humility and pride that we, the editors, extend our gratitude to our reviewers who dedicate their expertise and volunteer time to make EBR the best journal that it can be.

This year is the year we further enhance our access. Our presence will expand through social media that has been shown to be so robust in disseminating information and advancing citations for our authors. Our hope is to increase awareness of relevant articles and help clinicians treat patients with epilepsy and behavior consequences.

I am pleased to announce the selection of the EBR paper of the year 2019–2020 goes to Dr. A.A. Sharma et al. detailing “A preliminary study of the effects of cannabidiol (CBD) on brain structure in patients with epilepsy”. This is based upon multiple criteria including editor scores during the review process, number of citations and downloads, and the impact seen on social media.

To produce a top-ranked journal, there are multiple members of the team that are important in producing a well-designed product. Many thanks go to the editorial staff at Elsevier, Mehroon Farooqui, and executive publisher Annie Zhao.

As we enter 2021, we look forward to receiving more interesting manuscripts dealing with epilepsy and behavior. Thank you to our board members and reviewers. Thank you to our authors for terrific and interesting submissions and thank you to our readers for your interest in our journal reporting on rare, unexpected, and novel findings in people with epilepsy. From the editorial staff and on behalf of Elsevier, we wish you and yours health, happiness, and safety throughout 2021.

